# Characterizing Cellular Physiological States with Three-Dimensional Shape Descriptors for Cell Membranes

**DOI:** 10.3390/membranes14060137

**Published:** 2024-06-07

**Authors:** Guoye Guan, Yixuan Chen, Hongli Wang, Qi Ouyang, Chao Tang

**Affiliations:** 1 Center for Quantitative Biology, Peking University, Beijing 100871, China; guanguoye@gmail.com (G.G.); qoy@zju.edu.cn (Q.O.); 2 School of Physics, Peking University, Beijing 100871, China; yixuanchen@stu.pku.edu.cn; 3 School of Physics, Zhejiang University, Hangzhou 310027, China; 4 Peking-Tsinghua Center for Life Sciences, Peking University, Beijing 100871, China

**Keywords:** *Caenorhabditis elegans*, 3D shape descriptor, fluorescence imaging, cell membrane, cell division (cytokinesis), cell migration, cell lineage, cell fate, gene expression, embryogenesis

## Abstract

The shape of a cell as defined by its membrane can be closely associated with its physiological state. For example, the irregular shapes of cancerous cells and elongated shapes of neuron cells often reflect specific functions, such as cell motility and cell communication. However, it remains unclear whether and which cell shape descriptors can characterize different cellular physiological states. In this study, 12 geometric shape descriptors for a three-dimensional (3D) object were collected from the previous literature and tested with a public dataset of ~400,000 independent 3D cell regions segmented based on fluorescent labeling of the cell membranes in *Caenorhabditis elegans* embryos. It is revealed that those shape descriptors can faithfully characterize cellular physiological states, including (1) cell division (cytokinesis), along with an abrupt increase in the elongation ratio; (2) a negative correlation of cell migration speed with cell sphericity; (3) cell lineage specification with symmetrically patterned cell shape changes; and (4) cell fate specification with differential gene expression and differential cell shapes. The descriptors established may be used to identify and predict the diverse physiological states in numerous cells, which could be used for not only studying developmental morphogenesis but also diagnosing human disease (e.g., the rapid detection of abnormal cells).

## 1. Introduction

The shape of a cell, as defined by its membrane, is controlled by both intracellular and extracellular mechanics, as well as underlying molecular activities [1]. Therefore, it is closely related to the cellular physiological states in multiple dimensions. For example, changes occur in the mechanical properties of a cell during cell division (cytokinesis) and cell fate specification [2,3], and branch-shaped microglial cells are involved in setting up synaptic connectivity and responding to neuronal signals [4,5]. In the context of cells associated with disease, a well-known example is cancerous cells that often exhibit abnormally variable shapes, along with unlimited proliferation, invasive migration, and blocking of external signals [6,7]. However, systematic and analytical tools for linking cell (membrane) shapes with cellular physiological states are still lacking. Thus, it remains difficult to accurately predict whether a cell is undergoing a certain biological process only from its shape.

In recent years, an increasing number of advanced experimental and computational studies have successfully established high-quality datasets of cell shapes during embryonic development in animals such as nematodes, ascidians, fruit flies, and zebrafish [8,9,10]. Embryogenesis in such animals involves hundreds to thousands of cells that deform, migrate, divide, and differentiate drastically, providing informative resources for deciphering the interconnections between emerging cell shapes and cellular physiological states. Among the animal models mentioned above, the nematode *Caenorhabditis elegans* has been widely used in cell and developmental biology studies for roughly half a century, given its stereotypical and well-characterized developmental system which exhibits cell-level precision and robustness [11,12,13,14,15]. Recently, the early embryonic cell shapes of *C. elegans* have been evaluated to determine their influential factors, such as signaling between cells and the lifespans of cells [16,17].

In this study, we developed an integrated a computational framework that contains 12 3D shape descriptors derived from the literature for quantifying the shapes of cell membranes, implemented on a publicly available cell shape dataset of 17 *C. elegans* embryos which all cover the 4- to 350-cell stages (Figure S1) [18,19]. A total of four exemplary critical cellular physiological states were found to be characterizable: cell migration speed, cell division (cytokinesis), cell lineage specification, and gene expression specification. The methods and findings of this study offer a fresh perspective on the quantitative examination of cell shapes, which could enhance our understanding of cellular physiological states and their associated physical and biological processes. This could aid in furthering research on metazoan development and the diagnosis of diseases.

## 2. Material and Methods

### 2.1. Collection and Preprocessing of Digital Cell Shape Data from a Public Dataset

**Figure 1:**
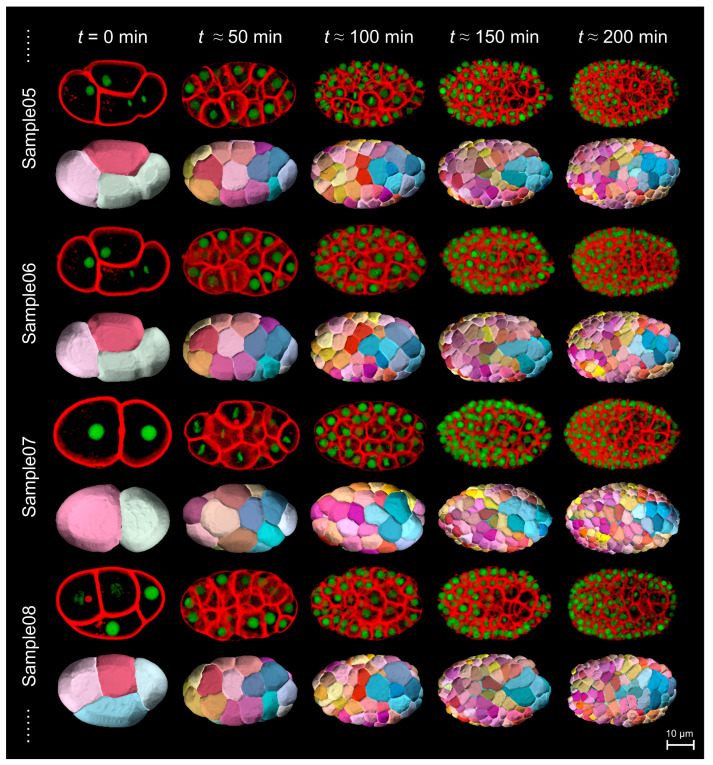
Fluorescence imaging and morphology reconstruction of wild-type *C. elegans* embryos (exemplified by Sample05–Sample08, from top to bottom) during imaging time t≈ 0–200 min, starting from no later than the 4-cell stage. For each embryo sample, the 3D projection of image stacks with GFP-labeled cell nuclei (green) and mCherry-labeled cell membranes (red) is shown in the upper row, while the automatic segmentation of cell membranes via *CShaper* is shown in the lower row [18,19]. Scale bar shown in the bottom right corner (10 μm). The relationships between cell identities and color maps are listed in Table S1. The corresponding time-lapse data are fully illustrated in Movie S1.

To aid the characterization of cellular physiological states by cell shape, we utilized our previously established dataset on 3D time-lapse cell shape reconstruction for the first half of *C. elegans* embryogenesis, in which all of the cells were unambiguously tracked and accompanied by quantitative information on their identity, fate, position, division timing, etc. (Figure S1) [18,19]. Confocal microscopy imaging was performed from no later than the 4–cell stage to beyond the 350–cell stage for 17 wild-type embryos (labeled Sample04–Sample20), whose cell nuclei were labeled with green fluorescent protein (GFP), and the cell membranes were labeled with mCherry (red) ubiquitously. While the cell nuclei were subjected to cell tracing and lineaging, an algorithm called *CShaper* was used to segment the cell membranes automatically, extracting the space of any cell enclosed by its corresponding membrane (Figure 1; Movie S1). In total, 381,781 final outputted cell shapes were generated at a temporal resolution of 1.39 min and a spatial resolution of 0.25 μm, with a 3D domain size of 184 × 256 × 114 pixels.

**Figure 2:**
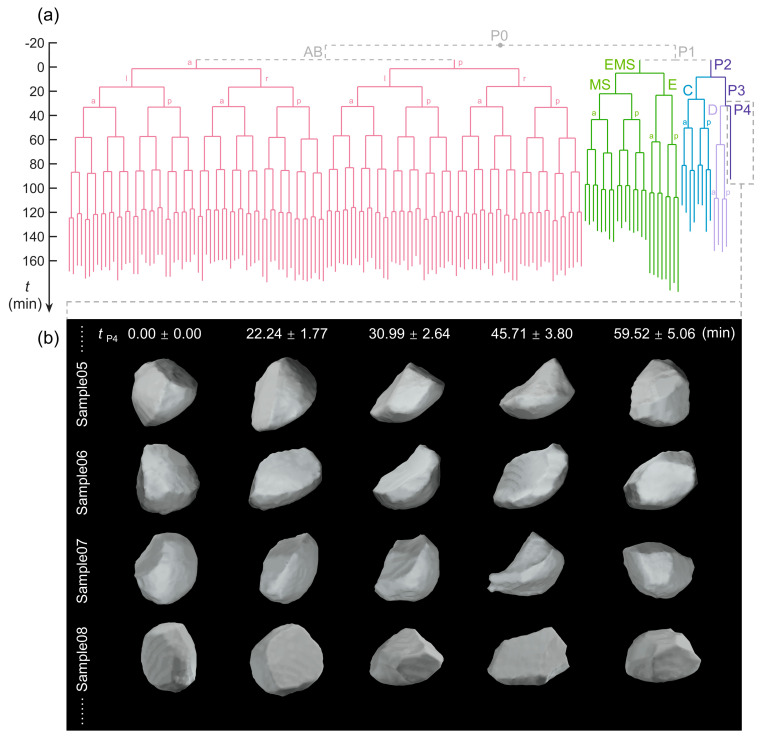
Cell lineage tree and cell shape data. (**a**) The cell lineage tree was averaged over 17 embryo samples, with the last moment of the 4–cell stage (ABa, ABp, EMS, and P2 cells) as time zero. The differentiated somatic cell lineages (AB, EMS, C, and D) and germline stem cells (P2, P3, and P4) are distinguished by different colors. The absent early cells beyond the imaging period are indicated by the gray dotted lines (P0, AB, and P1). (**b**) The 3D shape of the P4 cell in embryos of Sample05–Sample08 at specific time points (mean ± STD calculated with all of the 17 embryo samples). Note that $t_{P4}$ represents the actual lifespan of the P4 cell, with its birth considered as time zero and normalized over 17 embryo samples.

Next, we merged the original cell shape data of the 17 embryo samples by setting the last time point of the 4–cell stage as the starting moment and retaining only the cells for which a full lifespan (from newborn to division) was recorded and which were completely reproducible among all of the embryo samples. These were the AB4–AB128 cells (the 3rd–8th generations of the AB blastomere, primarily producing the epidermis, neurons, pharynx, and muscle), MS1–MS16 cells (the 1st–5th generations of the MS blastomere, primarily producing the pharynx and muscle), E1–E8 cells (the 1st–4th generations of the E blastomere, producing the gut), C1–C8 cells (the 1st–4th generations of the C blastomere, producing the epidermis, neurons, and muscle), D1–D4 cells (the 1st–3rd generations of the D blastomere, producing muscle), and P3 and P4 cells (producing germline) (Figure 2a) [15,20]. This step ensured that the resulting cell shape data for each cell and at each time point represented the conserved features among all of the embryo samples with high statistical reliability. Eventually, each cell within the average lineage tree had 17 independent groups of recorded cell shape data, which are exemplified by the shapes of the P4 cell in Figure 2b.

### 2.2. Shape Descriptors for a 3D Object

Twelve 3D shape descriptors with explicit geometrical significance were collected from the literature [21,22,23] and categorized into four groups: sphericity (general sphericity, diameter sphericity, intercept sphericity, and maximum projection sphericity), roundness (Hayakawa roundness), convex hull (spreading index), and shape factor (elongation ratio, pivotability index, Wilson flatness index, Hayakawa flatness ratio, Huang shape factor, and Corey shape factor). Their mathematical definition, geometric meaning (listed under Remarks), and relevant research are detailed in Table 1.

Before calculating the 3D shape descriptors above, several basic parameters needed to be prepared in advance, based on the 3D cell region made up of a cloud of pixels. (1) The surface area (S) was calculated as the sum of the areas of the triangles outputted by triangulation on a 3D cell region. (2) The volume (V) was calculated as the total amount of space enclosed by the boundary outputted by triangulation on a 3D cell region. (3) The surface area and volume ($S_{convex}$ and $V_{convex}$, respectively) of the convex hull enclosing a 3D cell region were calculated using the same methods as those used for S and V, respectively [24]. (4) The most commonly representative axes of an object are equivalent to those of its reference ellipsoid or the oriented bounding box (OBB) enclosing the 3D cell region, where a, b, and c denote the lengths of the long, intermediate, and short axes, respectively [25,26,27]. Principal component analysis (PCA) was implemented to calculate the triaxial orientation of the OBB with maximum variance in space, which corresponded to the eigenvectors of the covariance matrix:

(1) $$C=\left[\begin{array}{ccc} cov(X,X) & cov(X,Y) & cov(X,Z)\\ cov(Y,X) & cov(Y,Y) & cov(Y,Z)\\ cov(Z,X) & cov(Z,Y) & cov(Z,Z) \end{array}\right]$$

where X,Y,Z are the coordinates of all pixels in the x,y,z directions, respectively; exemplified by X and Y, $C_{i,j}=cov\left(X,Y\right)=\frac{1}{n-1}\sum_{i=1}^{n}\left(X_{i}-\bar{X})(Y_{i}-\bar{Y}\right)$ (n is the total pixel number and $\bar{X},\bar{Y}$ are the averages of X,Y respectively). Then, the projection of the 3D cell region onto each axis defined the values of a, b, and c. Based on the four groups of basic parameters, the 12 3D shape descriptors were calculated according to the mathematical definition in Table 1, in which the 3D cell regions with outstandingly large and small values for each descriptor are presented along with their triaxial lengths and corresponding orientations.

**Table 1:** The 3D shape descriptors for characterizing cell membranes.

Categorization	Shape Descriptor	Mathematical Definition	3D Cell Regions with Relatively Large (Top) and Small (Bottom) Values	Remarks	Relevant Research
Sphericity	General Sphericity	$\frac{\sqrt[{3}]{36\pi V^{2}}}{S}$	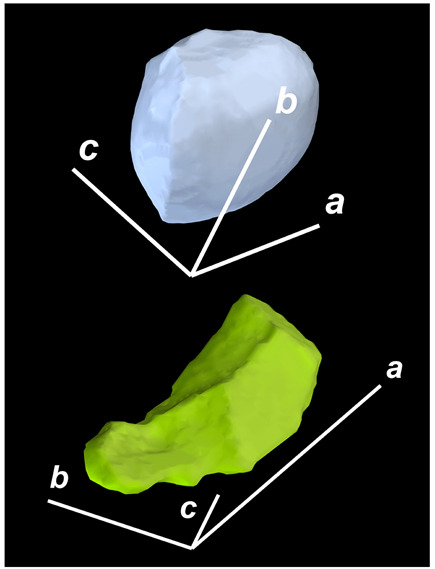	This formula was given in [28] and is the most generally used mathematical definition for describing the sphericity of a 3D object [25]. Thus, we call it “*general sphericity*” in this paper, while it is also called “*true sphericity*” in [28]. The ratio of the surface area of a perfect sphere having the same volume as the 3D object to the actual surface area of the 3D object [28]. This represents how similar the shape of a 3D object is to a perfect sphere [29].	[23,25,28,29,30,31,32,33,34,35]
	Diameter Sphericity	$\frac{\sqrt[{3}]{6V/\pi }}{a}$	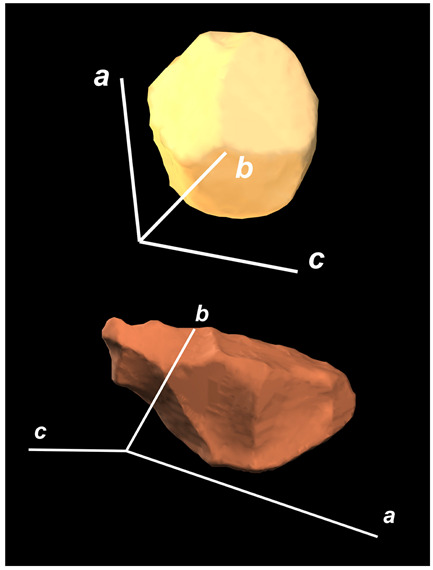	This formula was given in [36] and later termed by the authors of [31]. The ratio of the diameter of a perfect sphere having the same volume as the 3D object to the long axis of the 3D object [25]. This type of sphericity is used to describe the overall shape of the 3D object, irrespective of the sharpness of edges and corners [37].	[23,25,26,31,35,37,38]
	Intercept Sphericity	$\sqrt[{3}]{\frac{bc}{a^{2}}}$	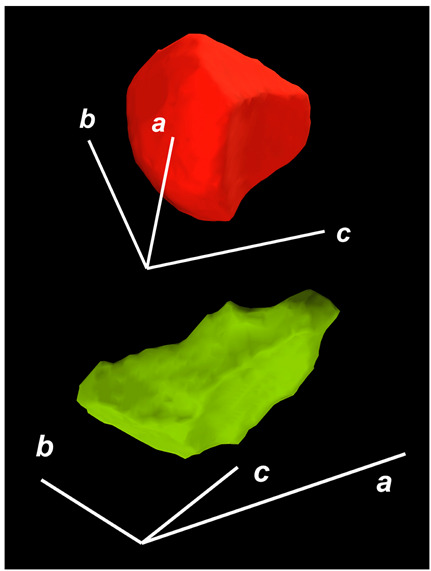	This formula was given in [26]. The volume ratio of the ellipsoid having a, b, and c as the lengths of the three axes to the circumscribing sphere having a as its diameter [25].	[22,23,25,26,31,32,38]
	Maximum Projection Sphericity	$\sqrt[{3}]{\frac{c^{2}}{ab}}$	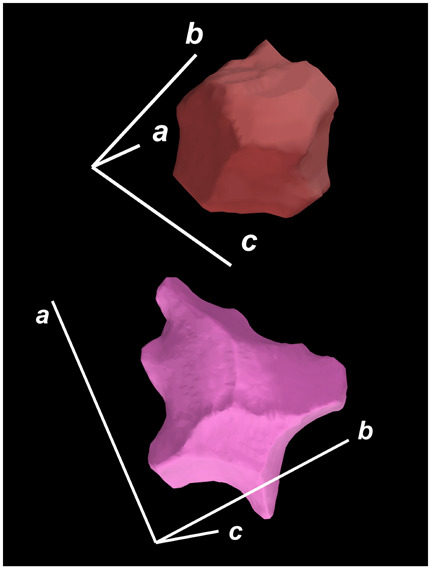	This formula was given in [38]. The ratio of the maximum projection area of a 3D object to the maximum projection area of the perfect sphere having the same volume as the 3D object [38].	[23,25,32,38]
Roundness	Hayakawa Roundness	$\frac{V}{S\sqrt[{3}]{abc}}$	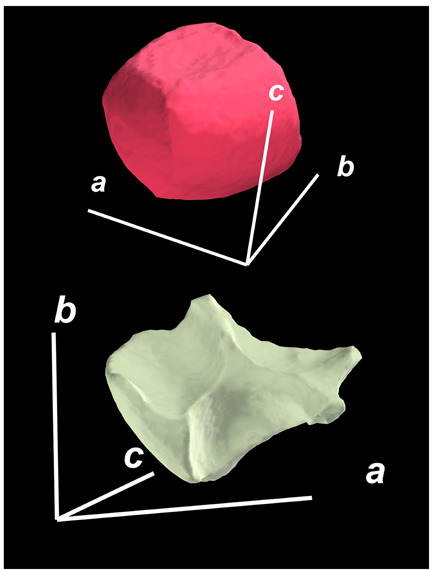	This formula was given in [23]. The difference between sphericity and roundness is that sphericity is a measure of the degree to which a 3D object approximates the shape of a perfect sphere and is independent of its size, while roundness is a measure of the sharpness of a 3D object’s edges and corners. Intuitively, they measure two different morphological properties; sphericity is essentially dependent on elongation, whereas roundness is essentially dependent on the sharpness of any angular protrusions (convexities) and indentations (concavities) on the surface of a 3D object [25]. As mentioned in [36], sphericity and roundness are different concepts, as the former represents the gross shape of a 3D object and the latter focuses on its edges and corners [23].	[23,25]
Convex Hull	Spreading Index	$\frac{\sqrt[{3}]{36\pi V_{convex}^{2}}}{S_{convex}}$	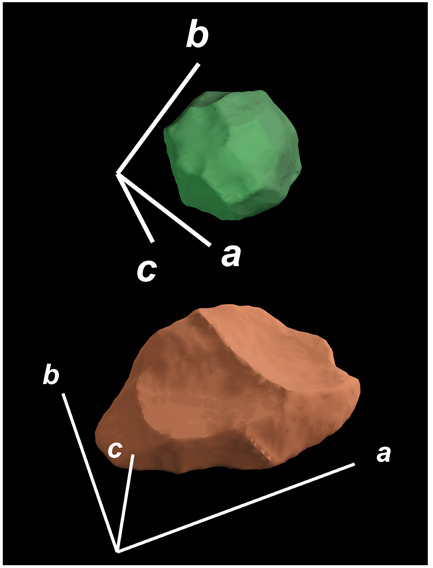	This formula was derived from the concept of the spreading index for a 2D object [21]. The spreading index is based on the convex area but could additionally measure the degree of roundness of the convex hull, thus reflecting the spreading of a 3D object, such as a cell [21].	[21,33,39]
Shape Factor	Elongation Ratio	$\frac{a}{b}$	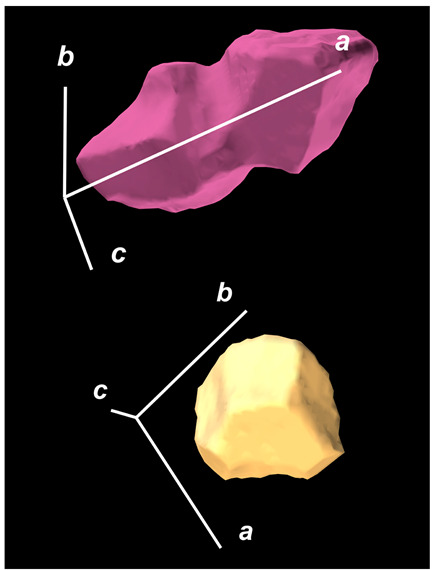	This formula was given in [40].	[22,23,25,26,30,32,40,41]
	Pivotability Index	$\frac{c}{b}$	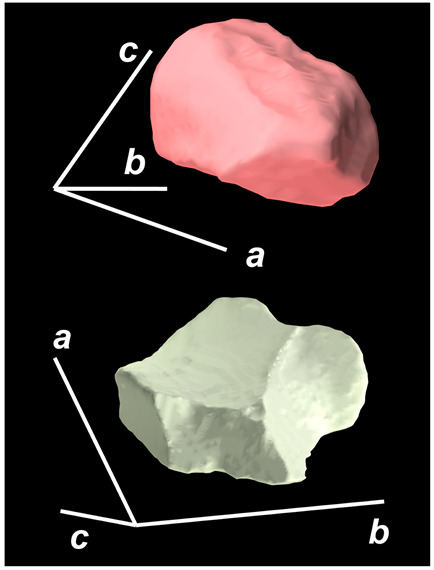	This formula was given in [40] and was also called the “*rollability index*” in [41].	[22,23,25,26,30,32,40,41]
	Wilson Flatness Index	$\frac{c}{a}$	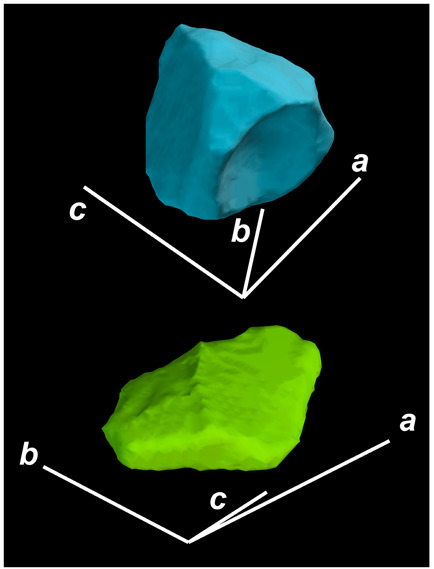	This formula was given in [22].	[22,25,41]
	Hayakawa Flatness Ratio	$\frac{a+b}{2c}$	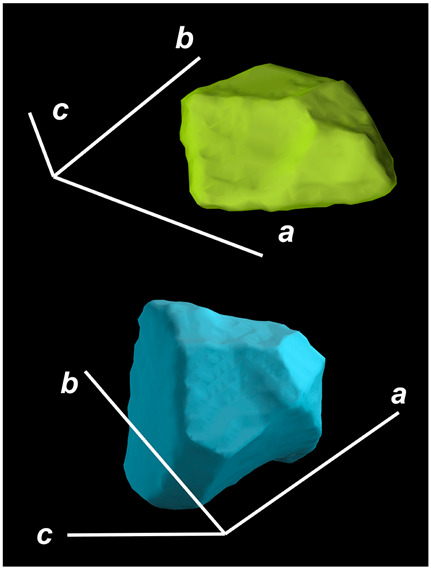	This formula was given in [23].	[23]
	Huang Shape Factor	$\frac{b+c}{2a}$	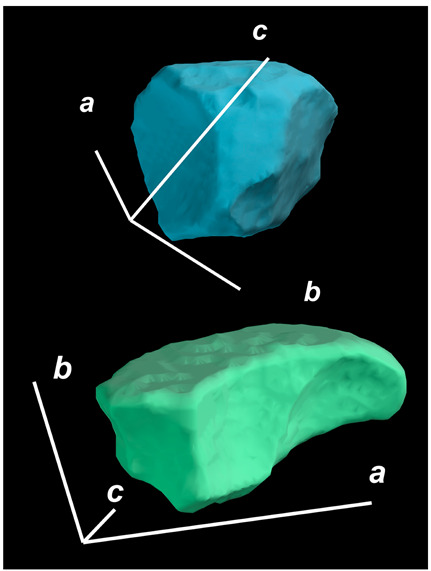	This formula was given in [22].	[22,32]
	Corey Shape Factor	$\frac{c}{\sqrt{ab}}$	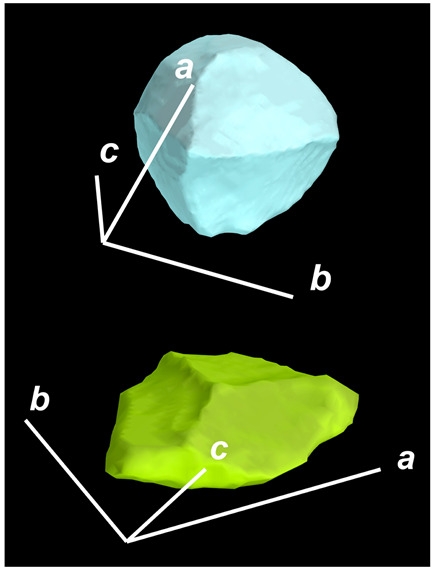	This formula was given in [22].	[22,23,32,41]

## 3. Results

### 3.1. Measurement Precision

Limited by the confocal microscopy and cell membrane segmentation algorithm, the digital 3D cell regions in the *CShaper* dataset have a recognized boundary with a pixel size thickness of 0.25 μm [18,19]. Concerning this uncertainty originating from microscopy and segmentation, for all of the 381,781 3D cell regions covering from the 2- to beyond 350−cell stages in *C. elegans* embryogenesis (Figure 1; Movie S1), we added (adding all non-cell pixels that had direct contact with a cell pixel) or removed (removing all cell pixels that had direct contact with a non-cell pixel) a one-pixel layer and calculated the new values of all 3D shape descriptors. For any specific 3D shape descriptor applied on a given 3D cell region, the original and new values are denoted by $\xi_{1}$, $\xi_{2}$ and $\xi_{3}$.

The precision of the 3D shape descriptors was estimated with two metrics, namely the coefficient of variation ($\eta_{1}$) and relative change ($\eta_{2}$) of $\left\{\xi_{i}\right\}\;\left(i=1,2,3\right)$:

(2) $$\eta_{1}=\frac{\sqrt{\frac{1}{3}\Sigma_{i=1}^{3}(\xi_{i}-\bar{\xi })}}{\bar{\xi }}$$

(3) $$\eta_{2}=\frac{\max\left(\left\{\xi_{i}\right\}\right)-min(\left\{\xi_{i}\right\})}{\bar{\xi }}$$

where $\bar{\xi }$ denotes the average of $\left\{\xi_{i}\right\}$. For each of the 12 3D shaper descriptors (Table 1), both $\eta_{1}$ and $\eta_{2}$ exhibited an average always smaller than 10%, suggesting considerable measurement precision in the case of the *CShaper* dataset used in this study (Table S2).

### 3.2. Characterization of Cytokinesis with the Elongation Ratio

Cytokinesis, namely the biological process of a mother cell dividing into two daughter cells with the separation of both the cell nuclei and membranes, is known to proceed with an elongated cell body aligned to separate properly into the two daughter cells [42]. According to prior knowledge in molecular and cellular biology, such a phenomenon usually involves actin bundle network reorganization driven by myosin motors, which position the contraction ring at the cell equator (i.e., the cleavage plane). Subsequently, the density of myosin motors around the contraction ring increases through self-recruitment, enhancing the contraction force to separate a mother cell into two daughter cells [43,44].

**Figure 3:**
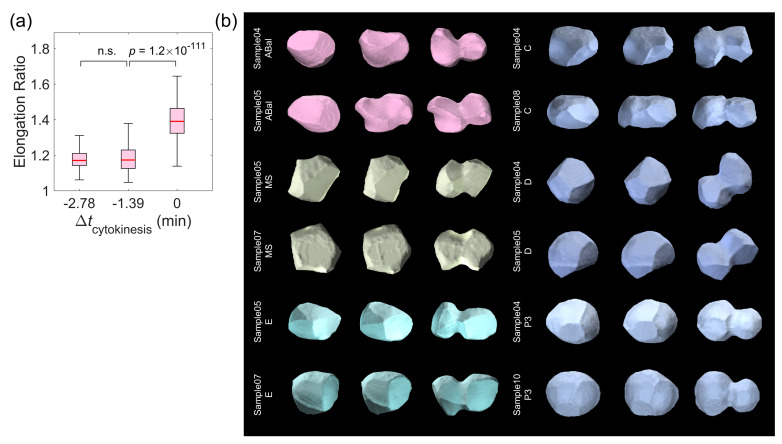
The elongation ratio significantly distinguishes cytokinesis during cell division. (**a**) The statistical comparison (two-sample *t*-test, where n.s. means not significant with a *p* value > 0.1) of the elongation ratio between the last three time points before the complete divisions of all cells, where $\Delta t_{cytokinesis}$ represents the time to the last time point. (**b**) The shape dynamics of cells from various lineages. For each cell shown in two embryo samples, the cell shapes at the last three time points before the complete divisions are listed from left to right.

Here, we applied the elongation ratio to the last three time points before the complete divisions of all 326 reproducible cells (Figure 2a) [23]. Notably, there was no significant difference between the first two time points (–2.78 and –1.39 min), but there was a significant increase in the elongation ratio at the last time point before complete cell division (from –1.39 to 0 min) (Figure 3a). Such shape dynamics with emerging cell body elongation coupled with a contractile ring in the cleavage plane can be intuitively visualized for cells from all lineages (i.e., AB, MS, E, C, D, and P), as shown in Figure 3b.

### 3.3. Negative Correlation between Cell Migration Speed and Sphericity

Cell migration speed, the quantitative measurement of cell motility, is a crucial measure, as cell migration takes place not only during normal development but also during cancer metastasis [45,46]. According to prior knowledge in molecular and cellular biology, such a phenomenon usually involves a decrease in overall cell cortical contractility and cell stiffness, along with a positive feedback loop between cortical flows and contractility gradients which establish an axis of cell polarity prior to cell migration. Such cell polarity maintained by actin cytoskeleton further induces cellular protrusions (i.e., pseudopodia) on a cell’s periphery, including sheet-like lamellipodia and needle-like filopodia formed by actin aggregation or contraction force-mediated membrane bleb [47,48].

**Figure 4:**
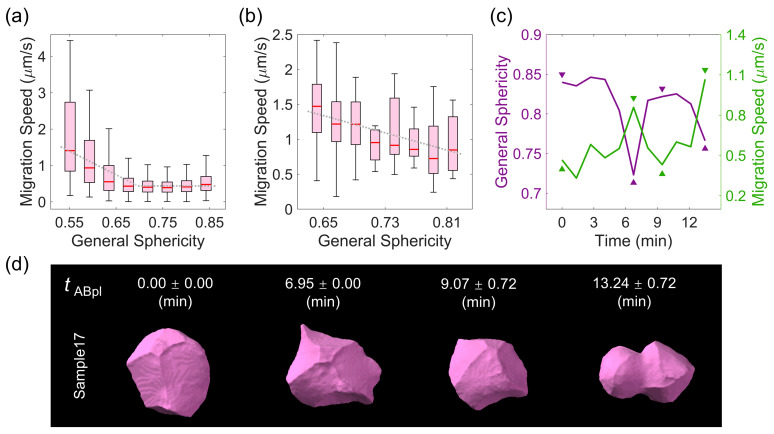
Negative correlation between cell migration speed and sphericity, represented by general sphericity. (**a**) The distribution of migration speed for all reproducible cells against their variable general sphericity. (**b**) The distribution of migration speed for the ABpl cell against its variable general sphericity. For (**a**,**b**), the plotted boxes were constructed using the data range from the lower quartile ($Q_{1}$) to the upper quartile ($Q_{3}$), with a line inside showing the median ($Q_{2}$) and the two bars showing the lower limit $[Q_{1}-1.5\left(Q_{3}-Q_{1}\right)$] and the upper limit $[Q_{3}+1.5\left(Q_{3}-Q_{1}\right)$]. (**c**) The change in general sphericity and migration speed in the normalized lifetime of the ABpl cell, averaged over all 17 embryo samples and with four opposite peaks indicated by triangles. (**d**) The 3D shape of the ABpl cell in embryo Sample17 at the time points (mean ± STD calculated using all 17 embryo samples) indicated in (**c**). Note that $t_{ABpl}$ represents the actual lifespan of the ABpl cell, with its birth considered as time zero and normalized over 17 embryo samples.

Although previous qualitative research [18,49,50] has proposed that some special cells, such as ABpl in *C. elegans* embryogenesis, have low sphericity and high motility, it is still unclear whether such a correlation between sphericity and motility can be generalized across all cells from the quantitative perspective. We noticed that the correlation between the cell migration speed (defined by the spatial deviation of the mass center of a cell between two consecutive time points) and general sphericity held when the general sphericity was smaller than 0.70 but not when it was larger than 0.70. This clear coupling and decoupling depending on the general sphericity suggests that cell migration was underway with the geometric changes, such as formation of the lamellipodia, protrusions, and filopodia [49], which may be predicted by the threshold of general sphericity (Figure 4a). As exemplified by the fast-migrating cell reported before, the data on ABpl yielded a correlation spanning all values of general sphericity, suggesting that cell migration is strictly controlled by a mechanism connecting it and cell sphericity (Figure 4b). An oscillation with two peaks and two valleys in both the general sphericity and cell migration speed further demonstrates such strong coupling over time. The four sets of extreme time points correspond to the birth of a cell, the middle of its lifespan, the moment near nucleus division, and the moment near membrane separation (with a high elongation ratio and low general sphericity) (Figure 4c). The visualized morphology of ABpl indicates that general sphericity approached the first peak when ABpl split from its mother cell ABp and the second peak when its nucleus was about to divide [17]. Intriguingly, the ABpl cell migrated long distances in the anterior or ventral direction during metaphase, along with several humps on the edges of the cell. When the cell membrane was approaching separation at the last time point, the decrease in sphericity was attributable to the extended cell membrane, in line with the increasing elongation ratio in Figure 3 (Figure 4d). It should also be pointed out that the general sphericity was found to be correlated with the other sphericity descriptors (diameter sphericity, intercept sphericity, and maximum projection sphericity), meaning that they were highly interchangeable despite having different mathematical definitions (Figure S2).

**Figure 5:**
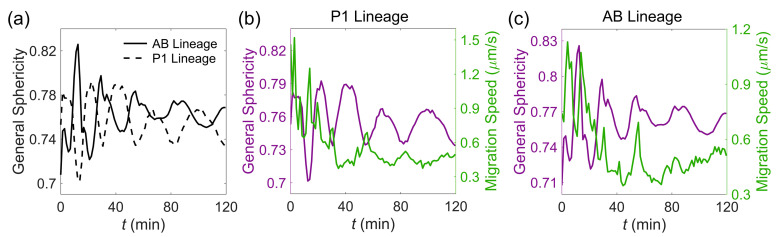
Oscillation in cell sphericity and migration speed. (**a**) The coupled oscillation of general sphericity in both the AB (solid) and P1 lineages (dashed) with opposite phases. (**b**) The coupled oscillation of general sphericity (left, purple) and migration speed (right, green) in the P1 lineage with opposite phases. (**c**) The decoupled oscillation of general sphericity (left, purple) and migration speed (right, green) in the AB lineage. For (**a**–**c**), the value on each curve was obtained by averaging those of all living cells in the targeted lineage and in all embryo samples at each time point.

Apart from changes in the cell migration speed demonstrated above, it was previously reported that an embryonic cell would become stiffer and more spherical near cell division and become softer and less spherical near the middle of its lifespan due to dynamic cytoskeleton remodeling [17,51]. Thus, we wondered whether two cell groups with differential division timings would have differential sphericity as well with respect to the development time. To this end, we considered the first two blastomeres derived by the division of the zygote—the AB and P1 cells—which have been shown to have differential proliferation rates in multiple generations [52,53]. Interestingly, the two cell groups exhibited oscillatory dynamics in cell sphericity, with highly persistent and opposite phases (Figure 5a). However, while the cell migration speed also oscillated against cell sphericity in the P1 lineage (Figure 5b), these parameters appeared to be decoupled in the AB lineage (Figure 5c), implying that more regulatory mechanisms were involved in a lineage-dependent manner.

### 3.4. Lineage-Dependent Differentiation of Cell Shape

**Figure 6:**
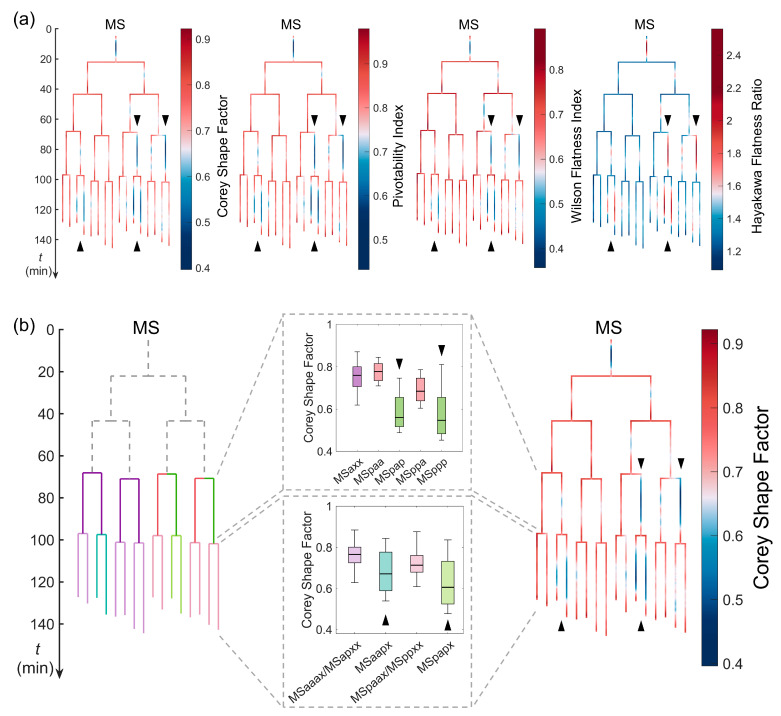
Lineage-dependent differentiation of cell shape, exemplified by the MS lineage. (**a**) The time-lapse distribution of the Corey shape factor (1st column), pivotability index (2nd column), Wilson flatness index (3rd column), and Hayakawa flatness ratio (4th column), shown with a colored tree. The MSpap, MSppp, MSaapx, and MSpapx cells are indicated by triangles to highlight their substantially smaller or larger values relative to others. (**b**) The substantially smaller Corey shape factor of the MSpap, MSppp, MSaapx, and MSpapx cells (indicated by triangles) relative to others. The plotted box was constructed using the data range from the lower quartile ($Q_{1}$) to the upper quartile ($Q_{3}$), with a line inside showing the median ($Q_{2}$) and the two bars showing the lower limit [$Q_{1}-1.5\left(Q_{3}-Q_{1}\right)$] and the upper limit $[Q_{3}+1.5\left(Q_{3}-Q_{1}\right)$].

Cell lineage is the history of an origin cell (e.g., zygote) proliferating into its offspring over generations, with differentiation occurring not only in cell fate but also in other cellular properties, such as cell size and cell cycle length [52,53]. However, it remains unclear whether cell shape is differentiated simultaneously as well, especially with respect to its explicit and interpretable geometrical features. To investigate this, we took the MS lineage, the anterior blastomere derived from the second somatic founder cell (i.e., EMS), as an example. In terms of its fourth and fifth generations, we derived four shape descriptors (i.e., the Corey shape factor, pivotability index, Wilson flatness index, and Hayakawa flatness ratio) from the literature, which exhibited substantially smaller or larger values in cells from different sublineages and in the symmetric (same) lineal position than their sisters or cousins (Figure 6a) but were found to be significantly correlated to one another (Figure S3). In particular, MSpxp (MSpap and MSppp) cells were the outliers in the MS8 cells, and MSxapx (MSaapa, MSaapp, MSpapa, and MSpapp) cells were the outliers in the MS16 cells. Interestingly, the cell shape differentiation in the fourth and fifth generations showed differences in symmetry with respect to the lineal positions of the cells. While the MSpxp was in the same lineal position within the MSpa and MSpp sublineages, the MSxapx was in the same lineal position within the MSa and MSp sublineages. Such switched cell shape symmetries during lineage development might contribute to the proper assembly of particular tissues and organs. Such cell shape differentiation can be inheritable (revealed by MSpap and its daughters MSpapx) and can also emerge (revealed by MSaap and its daughters MSaapx) or disappear (revealed by MSppp and its daughters MSpppx), leading to concordant separation and convergence of cell shapes during embryogenesis (Figure S4).

### 3.5. Simultaneous Differentiation of Cell Shape and Gene Expression

**Figure 7:**
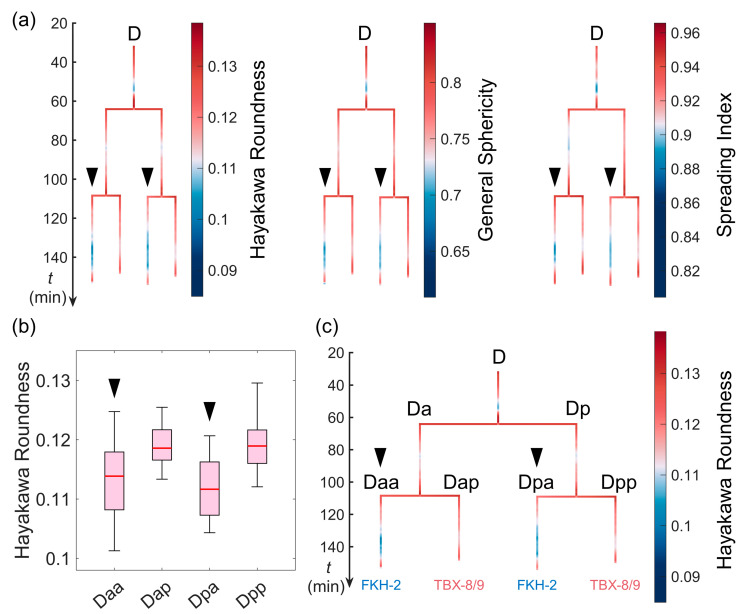
Simultaneous differentiation of cell shape and gene expression, exemplified by the D lineage. (**a**) The time-lapse distribution of Hayakawa roundness (1st column), general sphericity (2nd column), and the spreading index (3rd column) for cells within the D lineage shown with a colored tree. The Daa and Dpa cells are indicated by triangles to highlight their substantially smaller values relative to other cells. (**b**) The substantially smaller Hayakawa roundness values in the Daa and Dpa cells (indicated by triangles) relative to their sister cells. The plotted box was constructed using the data range from the lower quartile ($Q_{1}$) to the upper quartile ($Q_{3}$), with a line inside showing the median ($Q_{2}$) and two bars showing the lower limit $[Q_{1}-1.5\left(Q_{3}-Q_{1}\right)$] and the upper limit [$Q_{3}+1.5\left(Q_{3}-Q_{1}\right)$]. (**c**) In terms of the 3rd generation of the D lineage, the anterior (Daa and Dpa) and posterior (Dap and Dpp) cells had differential expression of FKH-2 and TBX-8/9, respectively, as revealed by previous experimental reports [60].

Cell fate is one of the most important cellular properties differentiated and diversified in embryonic development and is characterized by the differential expression of particular genes [15,54,55]. It is unclear whether cell shape differentiation can occur in line with cell differentiation at the molecular level. To investigate this, we took the D lineage, in which the blastomere is derived from the fourth somatic founder cell (i.e., D), as an example. Its third generation can be classified by three shape descriptors (i.e., the Hayakawa roundness, general sphericity, and the spreading index) that exhibit substantially smaller or larger values in cells from different sublineages but in the symmetric (same) lineal position, rather than their sisters or cousins (Figure 7a,b), but they were found to be significantly correlated to one another (Figure S5). In particular, Dxa (Daa and Dap) were the outliers in all D cells, although all of the terminal progeny within the D lineage differentiated into the body wall muscle without exception [15,56,57]. Fascinatingly, previous experimental reports demonstrated two transcription factors that show binary expression in line with the differentiated shapes of D cells. On the one side, TBX-8/9 (known to arrange the hypodermis and body wall muscle cell configuration spatially, where its absence leads to a disorganized morphogenetic structure [58]) was found to be expressed in Dxp but not in Dxa (Figure 7c). On the other side, FKH-2 (a neuroprotective gene, the inactivation of which aggravates motility defects and neurodegeneration and shortens lifespans [59]) was expressed in Dxa but not in Dxp. This consistency between differential cell shapes and differential gene expression suggests their potential coupling and interaction, which are worth investigating further.

### 3.6. User-Friendly Software for Calculating 3D Cell Shape Descriptors Automatically

**Figure 8:**
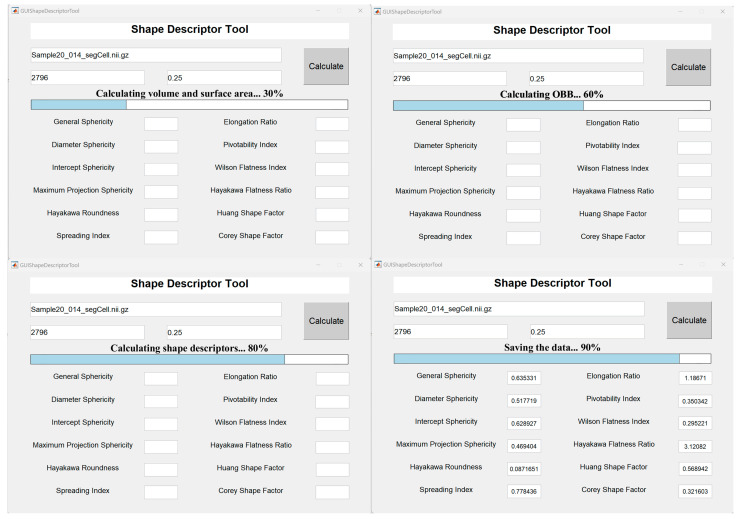
The graphical user interface of the *Shape Descriptor Tool* software. An exemplary case (the embryo Sample20, time point 14, ABpl cell) for calculating the 12 3D shape descriptors is shown. A part of the progress status with the corresponding running mission is listed as follows: the top left panel (30%), the top right panel (60%), the bottom left panel (80%), and the bottom right panel (90%).

In order to facilitate the convenient implementation and application of the 12 shape descriptors tested in this study, we constructed user-friendly software, named the *Shape Descriptor Tool*, based on *Matlab* (R2022b) (Figure S6) [61]. After inputting a 3D cell region digitized in a 3D matrix, the number label (or cell index) of the cell in it, and the spatial resolution (in μm), the values of all 12 3D shape descriptors could be calculated automatically with the progress shown on the interface (Figure 8). An instruction guidebook is provided in the Supplementary Text.

## 4. Discussion

Knowledge of the associations between cellular physiological states and cell shapes is of central importance not only for understanding the fundamental cell biology but also for providing the potential to develop effective methods for disease diagnosis, such as to detect cancerous cells [51,62]. In this study, we tested 12 quantitative 3D shape descriptors using a public dataset of cell morphology available for *C. elegans* embryogenesis by describing the cell shape dynamics in different cellular physiological states (Figure 1 and Figure 2; Table 1; Figure S1; Movie S1). While cell sphericity aand elongation ratio can uniquely predict cell division (cytokinesis) and cell migration speed, respectively (Figure 3 and Figure 4, respectively), multiple shape descriptors (e.g., the Corey shape factor and Hayakawa roundness) can identify the cell shape differentiation occurring simultaneously with the separation of cell lineage and gene expression (Figure 6 and Figure 7, respectively). The approaches and findings described in this paper provide new insights into the quantitative analysis of cell shape data for studying cellular physiological states and their underlying physical and biological mechanisms and may facilitate further research on metazoan development and disease diagnosis.

The applications of the approaches for and findings characterizing the cellular physiological states associated with cell shapes in this paper can be extended to many aspects:

- Regarding the specification of cell lineage and cell fate coupled with cell shape, systematic analysis of all cells and all stages throughout embryogenesis can be carried out beyond the representative studies of the MS and D lineages mentioned above (Figure 6 and Figure 7, respectively). Meanwhile, more public datasets, such as datasets on gene expression (measured by a fluorescence reporter and RNA sequencing) and chromatin accessibility, can be included [54,55,63] to systematically delineate how the developmental dynamics at the molecular scale affect those at the cellular scale which are depicted by different aspects of the cell shape, as well as those at higher scales such as tissue-, organ-, and embryo-scale morphogenesis.
- As the shape descriptors reported in this paper have explicit geometric significance and have been validated by specific physiological phenomena, they can be applied to the datasets of other organisms, such as ascidians, fruit flies, and zebrafish [9,10,64]. Moreover, they exhibit the potential to be applied to clinical data for fast disease diagnosis, for example, to identify cancerous cells that probably have low sphericity and high motility [6,7,62,65,66]. Such applications might also be employed at other biological scales, such as at the levels of cell nuclear shape and tissue or organ shape, for both fundamental research and disease diagnosis [67,68].
- Cell shape has been demonstrated to be an output of intracellular and intercellular mechanics [69]. Thus, with a focus on deciphering the underlying mechanical activities and interactions from cell shapes, the quantitative approaches and data provided in this paper can be utilized in future studies [70,71,72,73,74,75]. For instance, the stereotypical dumbbell shape before cell division could be utilized for analyzing the curvature and tension of the cell membrane [76,77]. Such inversely inferred mechanical properties or distributions can be further used for simulating real systems more comprehensively, thereby clearing the deck for model construction, virtual experimentation, and mechanism identification [78,79].
- Aside from the shape descriptors explored in this paper, other descriptors with explicit geometrical significance should be investigated in the future, such as the cell–cell interface curvature [73,80] and the numbers of vertexes, edges, and faces [81,82]. In addition, some shape descriptors with global information that enable consequent high-fidelity quantitative feature extraction with less information loss, such as shape entropy [35,41], the shape spectrum descriptor [83,84], spherical harmonics decomposition [16], and the voxel-based 3D Fourier transform descriptor [85], could be explored using data analysis methodologies such as principal component analysis and deep learning or artificial intelligence [16,85,86,87].

Although our study demonstrated the capability of a collection of 3D shape descriptors in characterizing cellular physiological states, the revealed correlation does not always imply causality. According to prior knowledge in molecular and cellular biology, the cell membrane architecture, composed of the cytoskeleton, lipid, proteins related to adhesion and junction, and so forth, can be affected by a number of intrinsic and extrinsic factors, like intracellular metabolism and extracellular signaling [88,89,90]. For the cases involved in this study, while many molecular activities underlying cell division (characterized by the elongation ratio) and cell motility (characterized by the sphericity), including both the pool of cytoskeleton components as well as their interactions and dynamics, have been documented [91,92], the ones connecting patterned cell shape features to the differential cell lineage (characterized by the Corey shape factor, among other descriptors) and cell fate (characterized by the Hayakawa roundness, among other descriptors) through specific genes are still elusive, as exemplified by the MS and D cells evidenced in this study. Despite all of this, the tested multidimensional cell shape descriptors can be interpreted as a novel type of single-cell omics data comparable to those at the molecular scale, represented by the transcriptome and proteome [55,60]. It is worth carrying out combinatorial discovery in the future which incorporates all of these single-cell omics data and uncovers the comprehensive molecular blueprint of shape control in both cellular and multicellular circumstances.

## Data Availability

Both the original *CShaper* dataset used in this study and the final computational results of the 12 3D shape descriptors for all 3D cell regions, as well as the corresponding *Matlab* codes and *Shape Descriptor Tool* software, are available in the *Zenodo* repository at https://doi.org/10.5281/zenodo.11103327.

## Supplementary Materials

The following supporting information can be downloaded at https://www.mdpi.com/article/10.3390/membranes14060137/s1. Figure S1: The step-by-step instructions for accessing the previously published *CShaper* dataset with 3D cell regions of 17 *C. elegans* embryos (Sample04–Sample20), illustrated with website snapshots. (a) Access the official website of the original literature [18]. (b) Access the link in the “*Data availability*” section. (c) Access the link provided in the *figshare* repository. (d) Access the “*Segmentation Results*” folder in the *OneDrive* repository. (e) ① The “*RawData*” folder contains the raw fluorescence images with GFP-labeled cell nuclei and mCherry-labeled cell membranes. ② The “*SegmentedCell\Sample*_LabelUnified*” subfolder contains the segmentation results (i.e., the 3D cell regions). ③ The “*name_dictionary.csv*” file contains the relationship between cell identities and their corresponding number labels used in the segmentation results. Figure S2: Pairwise correlation between general sphericity with (a) diameter sphericity (R=0.7197), (b) intercept sphericity (R=0.5911), and (c) maximum projection sphericity (R=0.5616), where R denotes the Pearson correlation coefficient. Figure S3: Pairwise correlation between the Corey shape factor and the (a) Hayakawa flatness ratio, (b) pivotability index, and (c) Wilson flatness index, where R denotes the Pearson correlation coefficient. Figure S4: Change in the Corey shape factor of the MSa (1st column), MSpa (2nd column), and MSpp (3rd column) sublineages averaged over all 17 embryo samples. The names of cells are indicated by arrows, and cells with substantially smaller and larger values are indicated by blue and red lines, respectively. Figure S5: Pairwise correlation of Hayakawa roundness with (a) general sphericity and (b) the spreading index, where R denotes the Pearson correlation coefficient. Figure S6: The step-by-step instructions for using the *Shape Descriptor Tool* software, exemplified by the ABpl cell labeled with the number 2796, in the embryo Sample20 and at time point 14. (a) ① Open *Matlab* under the path of the “*GUIScript*” folder and double-click “*GUIShapeDescriptorTool.m*”. ② Click “*Run*” to initiate the software. ③ Input the file path and parameters (left: number label of the cell to be analyzed; right: spatial resolution in μm) in the interface, and then click “*Calculate*”. (b) The progress bar shows the running progress. (c) Once completed, the 12 shape descriptors are shown on the interface, with a .csv file automatically saved in the current folder. (d) In the output file, the first row stores the number label or cell index of the cell analyzed, followed by the names of the 12 shape descriptors as well as their corresponding values below. Table S1: The RGB code for coloring the *C. elegans* embryonic cells in Figure 1 and Movie S1. Table S2: The correlation coefficient and relative change in the 3D shape descriptors, estimated by 3D cell region boundary perturbation. Movie S1: The fluorescence image (top) and its corresponding membrane segmentation results (bottom) of Sample05–Sample08.
