# Supplementary material for: Characterizing Cellular Physiological States with Three-Dimensional Shape Descriptors for Cell Membranes
Source: Membranes (Basel). 2024 Jun 7;14(6):137. doi: 10.3390/membranes14060137 (PMC11205511; doi:10.3390/membranes14060137)
Supplement: Supplementary file 1 [file membranes-14-00137-s001.zip › membranes-2970671 -Supplementary Material.pdf]

Supplementary Materials:
Characterizing multidimensional cellular physiological states with quantitative 3D shape descriptors for cell membranes

Guoye Guan [1,†,§,#], Yixuan Chen [2,†], Hongli Wang [1,2,*], Qi Ouyang [1,2,3], Chao Tang [1,2,4,*]

1. Center for Quantitative Biology, Peking University, Beijing, China; guanguoye@gmail.com (G.G.); qoy@zju.edu.cn (Q.O.)
2. School of Physics, Peking University, Beijing, China; yixuanchen@stu.pku.edu.cn (Y.C.)
3. School of Physics, Zhejiang University, Hangzhou, China
4. Peking-Tsinghua Center for Life Sciences, Peking University, Beijing, China

§ Current address: Department of Systems Biology, Harvard Medical School, Boston, USA

# Current address: Department of Data Science, Dana-Farber Cancer Institute, Boston, USA

† These authors contributed equally to this work.

* Correspondence: hlwang@pku.edu.cn (H.W.); tangc@pku.edu.cn (C.T.)

Supplementary Figure

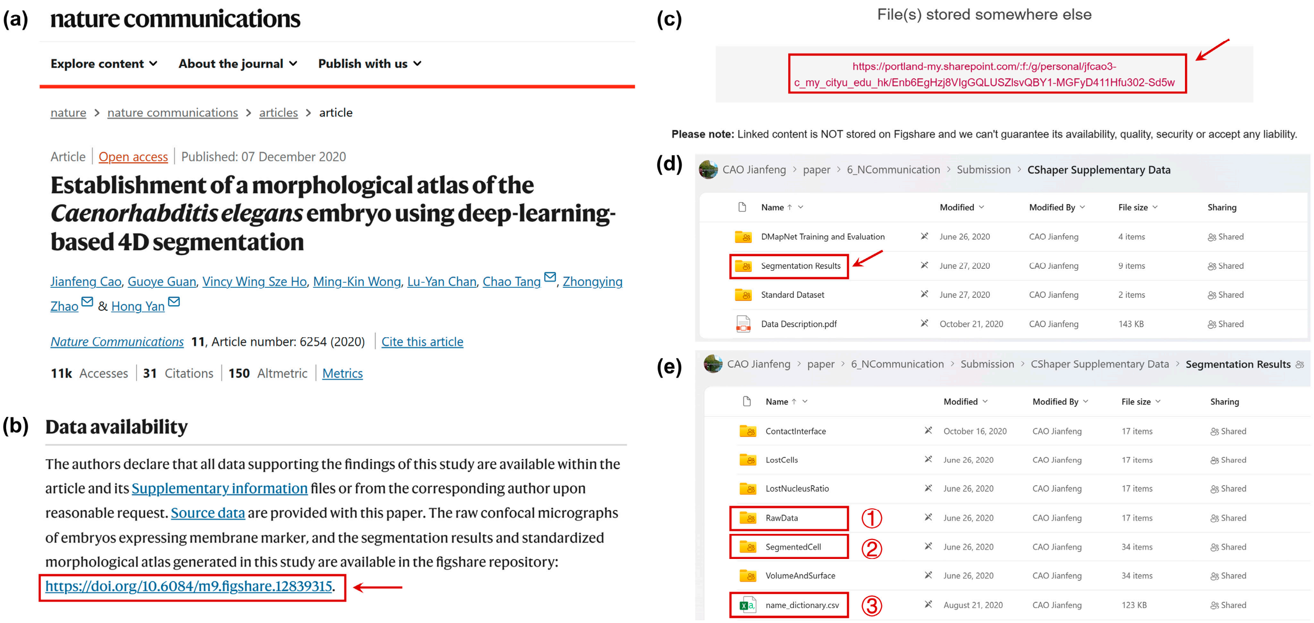

Figure S1. The step-by-step instruction for accessing the previously published *CShaper* dataset with 3D cell regions of 17 *C. elegans* embryos (Sample04−Sample20), illustrated with website snapshots. (a) Access the official website of the original literature [18]. (b) Access the link in the "*Data availability*" section. (c) Access the link provided in the *figshare* repository. (d) Access the "*Segmentation Results*" folder in the *OneDrive* repository. (e) ① The "*RawData*" folder contains the raw fluorescence images with GFP-labeled

cell nuclei and mCherry-labeled cell membranes. ② The "*SegmentedCell\Sample*_LabelUnified*" subfolder contains the segmentation results (*i.e.*, the 3D cell regions). ③ The "*name_dictionary.csv*" file contains the relationship between cell identities and their corresponding number labels used in the segmentation results.

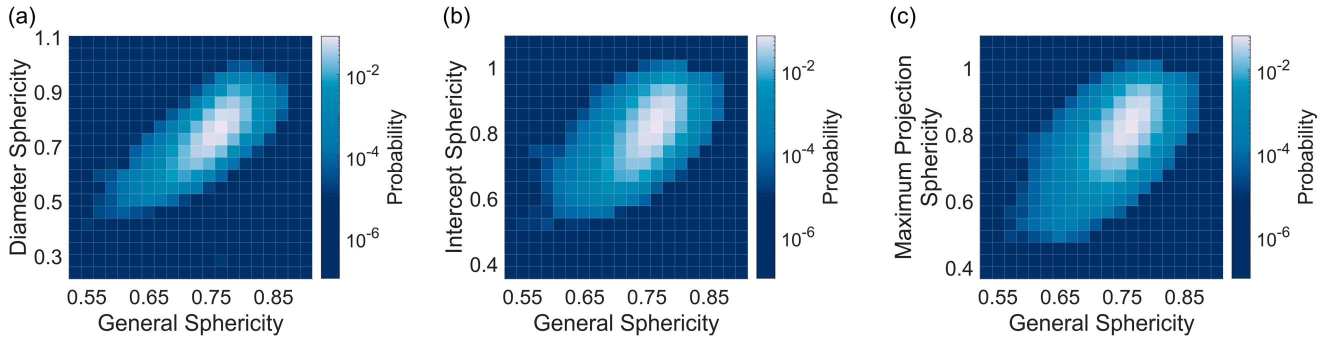

Figure S2. Pairwise correlation between general sphericity with (a) diameter sphericity ($R = 0.7197$), (b) intercept sphericity ($R = 0.5911$), and (c) maximum projection sphericity ($R = 0.5616$), where R denotes the Pearson correlation coefficient.

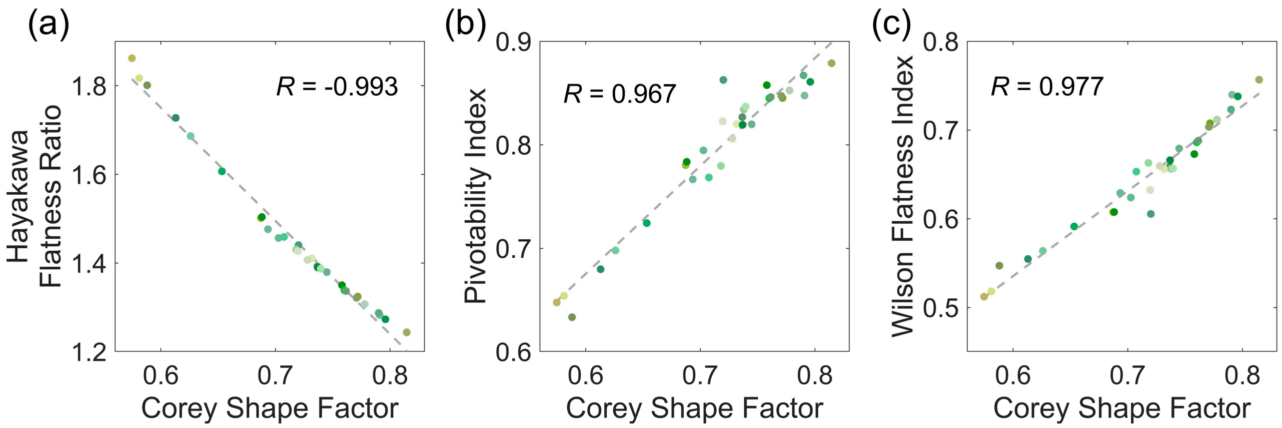

Figure S3. Pairwise correlation between the Corey shape factor and the (a) Hayakawa flatness ratio, (b) pivotability index, and (c) Wilson flatness index, where R denotes the Pearson correlation coefficient.

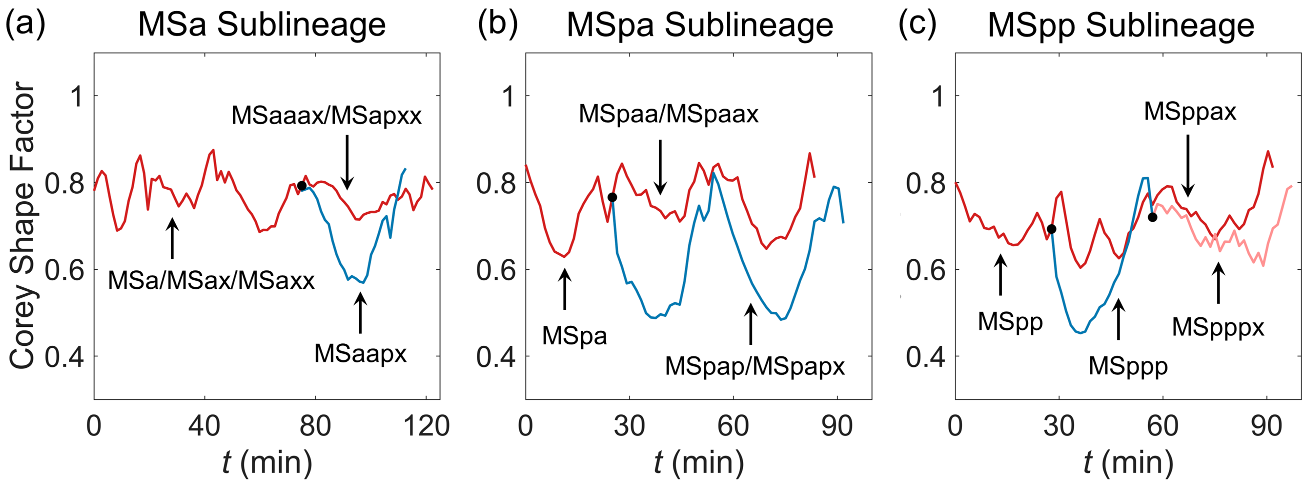

Figure S4. Change in the Corey shape factor of the MSa (1st column), MSpa (2nd column), and MSpp (3rd column) sublineages averaged over all 17 embryo samples. The names of cells are indicated by arrows, and cells with substantially smaller and larger values are indicated by blue and red lines, respectively.

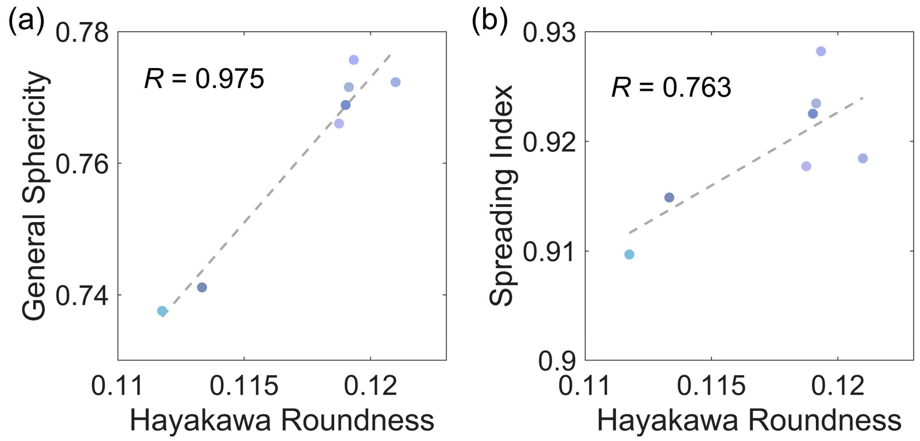

Figure S5. Pairwise correlation of Hayakawa roundness with (a) general sphericity and (b) the spreading index, where R denotes the Pearson correlation coefficient.

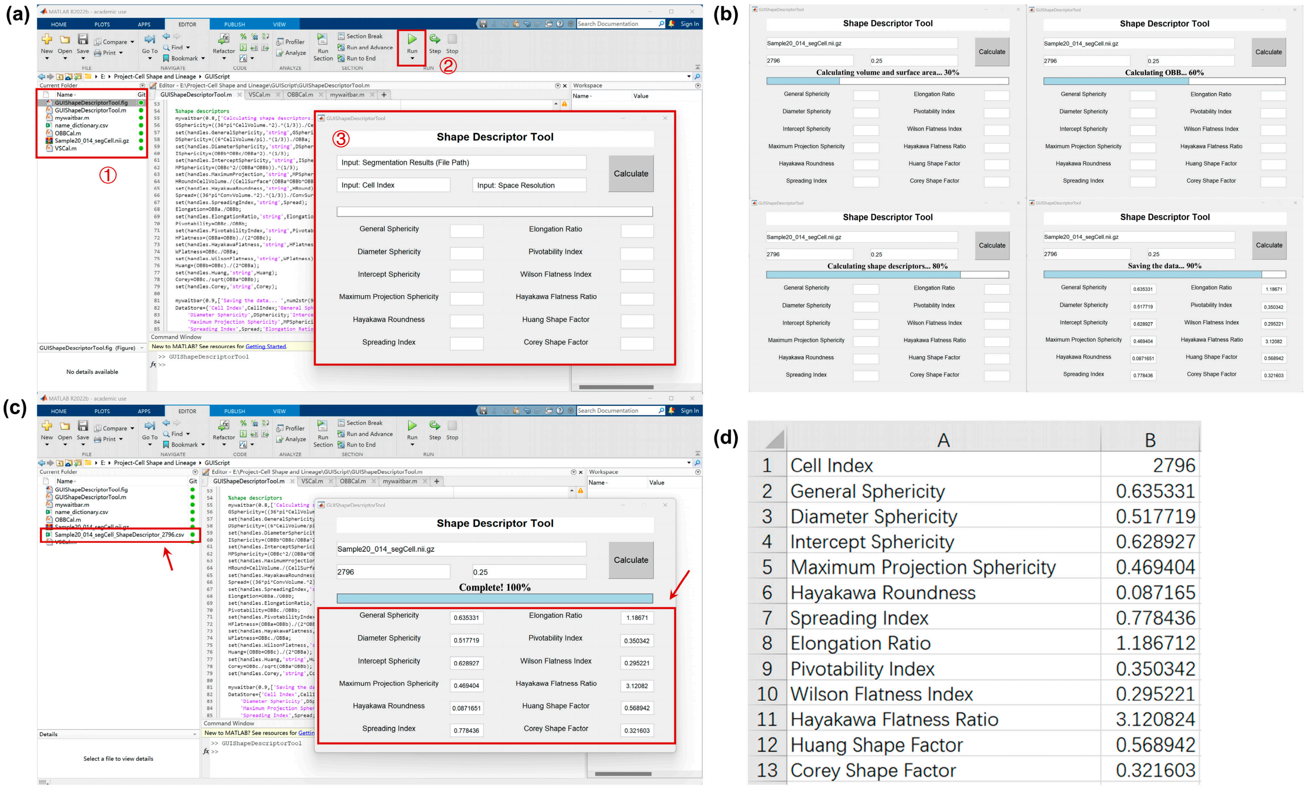

Figure S6. The step-by-step instruction for using the *Shape Descriptor Tool* software, exemplified by the ABpl cell labeled with the number 2796, in the embryo Sample20 and at time point 14. (a) ① Open *Matlab* under the path of the "*GUIScript*" folder and double-click "*GUIShapeDescriptorTool.m*". ② Click "*Run*" to initiate the software. ③ Input the file path and parameters (left: number label of the cell to be analyzed; right: spatial resolution in μm) in the interface, and then click "*Calculate*". (b) The progress bar shows the running progress. (c) Once completed, the 12 shape descriptors are shown on the interface, with a .csv file automatically saved in the current folder. (d) In the output file, the first row stores the number label or cell index of the cell analyzed, followed by the names of the 12 shape descriptors as well as their corresponding values below.

Supplementary Table

Table S1. The RGB code for coloring the *Caenorhabditis elegans* embryonic cells in Figure 1 and Movie 1.

Table S2. The correlation coefficient and relative change in the calculated 3D shape descriptors, estimated by 3D cell region boundary perturbation.

Supplementary Movie

Movie S1. The fluorescence image (top) and its corresponding membrane segmentation results (bottom) of Sample05−Sample08.

Supplementary Text

Instruction Guidebook for *Shape Descriptor Tool*

1. Introduction

The *Shape Descriptor Tool* software is a graphical user interface (GUI) constructed based on *Matlab* 2022b [61] for calculating the 12 3D shape descriptors of a given 3D cell region, as described in Section 2.2 of this article. All the computations were tested with a 12th Gen Intel(R) Core(TM) i7-1260P CPU.

2. Tutorial

The ABpl cell labeled with the number 2796, in the embryo Sample20 and at the time point 14, is an exemplary 3D cell region with an outstandingly small value in maximum projection sphericity as illustrated in Table 1, and is used for calculating the 12 shape descriptors here.

A. Download the "*GUIScript*" folder from https://doi.org/10.5281/zenodo.11103327. Inside, the "*Sample20_014_segCell.nii.gz*" file is the segmentation results of the embryo Sample20 at the time point 14, containing the ABpl cell labeled with the number 2796 and at the 7-cell stage.

B. Open *Matlab* under the "*GUIScript*" folder path and execute the "*GUIShapeDescriptorTool.m*" script. Click "*Run*" and then an interactive interface pops up (Figure S6a).

C. With the following information inputted, the GUI gives out the calculation results of the 12 shape descriptors on the interface.

   (1) Input "*Sample20_014_segCell.nii.gz*" into the "*Input: Segmentation Results (File Path)*". Change it to any desired file path, and ensure the input data is in the .nii.gz format.

   (2) According to the "*name_dictionary.csv*" file, the corresponding number label of the ABpl cell is 2796; thus, input "*2796*" into the "*Input: Cell Index*" box. Any number label or cell index is allowed so long as the corresponding cell exists in the segmentation file.

   (3) Input "*0.25*" into the "*Input: Space Resolution*" box, since the digital 3D cell regions in the *CShaper* dataset have a spatial resolution of 0.25 μm.

D. Click "*Run*" on the interface, and then its status is shown on the progress bar (Figure S6b), and the 12 shape descriptors are calculated and shown on the interface (Figure S6c).

E. A file named "*Sample20_014_segCell_ShapeDescriptor_2796.csv*" is generated under the same folder path as "*Sample20_014_segCell.nii.gz*" to store the output data, which contains both the cell index and 12 shape descriptors (Figure S6d). Here, the prefix "*Sample20_014_segCell*" is inherited from the input file, while the suffix "*2796*" is the number label or cell index of the ABpl cell.

3. Contact Information

All the scripts of the *Shape Descriptor Tool* software have been uploaded onto https://doi.org/10.5281/zenodo.11103327. If there is any question, please contact Guoye Guan (guanguoye@gmail.com) or Yixuan Chen (yixuanchen@stu.pku.edu.cn) anytime.
